# SMURF1/2 Are Novel Regulators of WNK1 Stability

**DOI:** 10.3390/kinasesphosphatases2030019

**Published:** 2024-09-20

**Authors:** Ankita B. Jaykumar, Sakina Plumber, Derk Binns, Chonlarat Wichaidit, Katherine Luby-Phelps, Melanie H. Cobb

**Affiliations:** 1 Department of Pharmacology, UT Southwestern Medical Center, Dallas, TX 75390, USA; 2 Department of Cell Biology, UT Southwestern Medical Center, Dallas, TX 75390, USA

**Keywords:** WNK1, OSR1, SPAK, SMURF1/2, ALK5

## Abstract

Angiogenesis is essential for remodeling and repairing existing vessels, and this process requires signaling pathways including those controlled by transforming growth factor beta (TGF-β). We have previously reported crosstalk between TGF-β and the protein kinase With No lysine (K) 1 (WNK1). Homozygous disruption of the gene encoding WNK1 results in lethality in mice near embryonic day E12 due to impaired angiogenesis, and this defect can be rescued by the endothelial-specific expression of an activated form of the WNK1 substrate kinase Oxidative Stress-Responsive 1 (OSR1). However, molecular processes regulated via a collaboration between TGF-β and WNK1/OSR1 are not well understood. Here, we show that WNK1 interacts with the E3 ubiquitin ligases SMURF1/2. In addition, we discovered that WNK1 regulates SMURF1/2 protein stability and vice versa. We also demonstrate that WNK1 activity regulates TGF-β receptor levels, in turn, controlling TGF-β signaling.

## 1. Introduction

Transforming growth factor beta (TGF-β) signaling modulates vascular remodeling [1]. Homozygous disruption of the gene encoding the protein kinase With No lysine (K) 1 (WNK1) results in defective angiogenesis around embryonic day E12 [2,3]. WNK1 is the most ubiquitously expressed among a family of four related atypical protein-serine/threonine kinases, known for their unique catalytic lysine site [4]. WNK1 binds and catalyzes the phosphorylation of substrates, Oxidative Stress Responsive 1 (OSR1) and STE20/SPS–1-related proline-alanine-rich kinase (SPAK, *STK39*) [5–9]. We previously reported crosstalk among WNK1 and multiple molecules involved in regulating TGF-β signaling pathways [10,11].

SMURF (SMAD Ubiquitination Regulatory Factor) 1/2 are WW-domain-containing enzymes belonging to the NEDD4 (Neural precursor cell Expressed, Developmentally Down-regulated 4) family of E3 ubiquitin ligases. SMURFs mediate endothelial–mesenchymal transition during cardiovascular development [12–20]. SMURF1 controls cellular responsiveness to the TGF-β/SMAD2 pathway [20]. TGF-β initiates redistribution of the TGF-β receptor type II into tight junctions which leads to the recruitment of SMURF 1 to cause the disintegration of tight junctions, a process critical to regulating cell migration and angiogenesis [21,22]. SMURF2, on the other hand, downregulates TGF-β signaling by targeting itself, SMAD2/3 transcription factors and the TGF-β receptor I, also known as Activin receptor-like kinase 5 (ALK5), for degradation [12–17,20,23,24]. This process is critical to regulating TGF-β signaling-dependent processes such as cell migration and angiogenesis [12–20,25]. Previous work from our laboratory showed that WNK1 is involved in the TGF-β pathway-dependent modulation of SMAD2/3 protein stability [10,11]. However, mechanisms underlying how WNK1 modulates TGF-β signaling and processes that, in turn, regulate WNK1 stability during TGF-β signaling are unknown.

Occludin is a tight junction protein which is important for regulating tight junction integrity [23,24,26]. Furthermore, recent studies show that occludin is also involved in endothelial vascular remodeling and angiogenesis [27]. We previously found that occludin interacts with OSR1 to enable tight junction turnover in a WNK1-dependent manner [10]. Our findings suggested intimate connections between TGF-β pathway molecules with the WNK1/OSR1 pathway to control angiogenesis. These findings lead us to speculate that WNK1 may modulate several TGF-β-dependent functions and that the regulation of WNK1 protein stability is an important determinant of TGF-β signaling output.

WNK1 protein turnover is regulated by several E3 ligases. WNK1 undergoes ubiquitination followed by degradation through the proteasome upon recruitment to the cullin3 (CUL3)-containing E3 ligase complex [28]. Another study from our group reported that ubiquitin-protein ligase E3 component n-recognin 5 (UBR5) interacts with WNK1 and its deficiency results in increased WNK1 protein [29]. A previous study showed that WNK1 isoforms are substrates of NEDD4–2, another member of the NEDD4 family of E3 ubiquitin ligase [30]. WNK1 binds to and phosphorylates NEDD4–2 [25,31,32]. NEDD4–2 negatively regulates TGF-β signaling by the ubiquitin-mediated degradation of SMAD2 and TGF-β type I receptor [33]. Therefore, the regulation of NEDD4–2 by WNK1 may also be involved in precisely controlling TGF-β signaling output to modulate cellular processes underlying angiogenesis. This led us to speculate that there may be other E3 ligases involved in the TGF-β signaling pathway that regulate WNK1 protein stability.

In this study, we identify molecular events that underlie WNK1-dependent inputs to the specificity of the TGF-β pathway in endothelial cells. We show interactions between WNK1 and SMURF1/2 and report that WNK1 forms discrete signaling microdomains (sometimes referred to as WNK bodies [25]) for the reciprocal regulation of WNK1 and E3 ubiquitin ligase SMURF1/2 turnover. In addition, we discover complex inter-regulation between WNK1 and SMURFs during TGF-β signaling. These findings add to the conclusion that WNK1 collaborates with downstream TGF-β signaling components SMURF1/2 to regulate and fine-tune processes involved in TGF-β signaling such as the turnover of TGF-β receptors in a context-dependent manner.

## 2. Results

### 2.1. WNK1 Colocalizes with SMURF1/2

**Figure 1.**
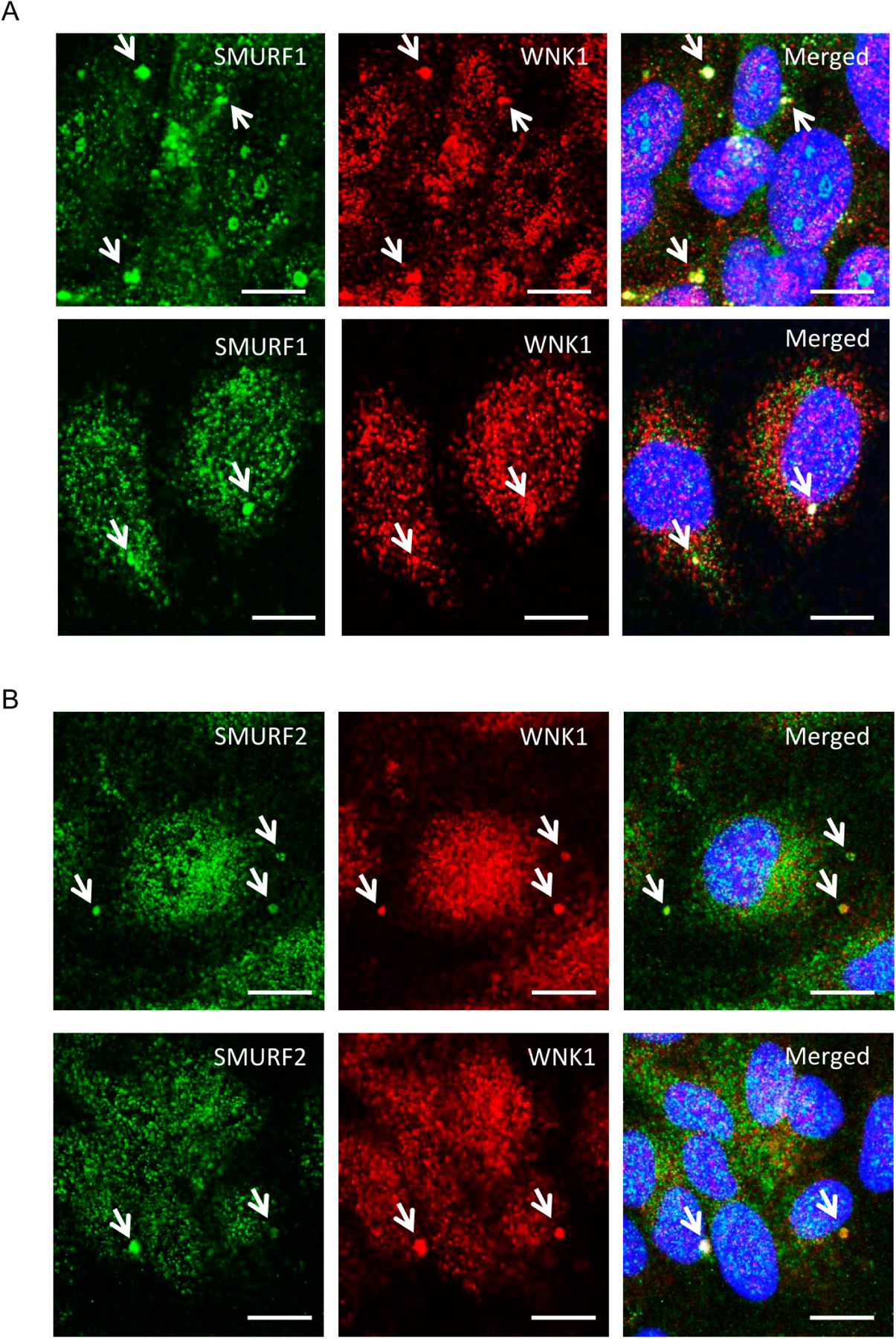
(**A**,**B**) Representative confocal images of immuno-fluorescently labeled endogenous WNK1 (red), SMURF1/2 (green) and nucleus (DAPI: blue) in primary HUVECs upon 1–2 h TGF-β (10 ng/mL) stimulation. Merged panel (yellow) shows co-localization between WNK1 and SMURF1/2 in large punctate structures. Scale bar = 20 μm; *n* = 3.

Given the involvement of WNK1 in TGF-β-SMAD signaling [10,11], we examined actions of WNK1 on TGF-β-pathway-dependent functions of SMURF1/2 in endothelial cells. First, we tested whether either SMURF1 or 2 co-localize with WNK1 in primary human umbilical vein endothelial cells (HUVECs). Interestingly, we found that a fraction of WNK1 and SMURF1 as well as SMURF2 were observed in large punctate structures distinct from their distributions elsewhere in cells [19] (Figure 1A,B).

### 2.2. WNK1 Kinase Activity Regulates Association of WNK1/OSR1 with SMURF2

**Figure 2.**
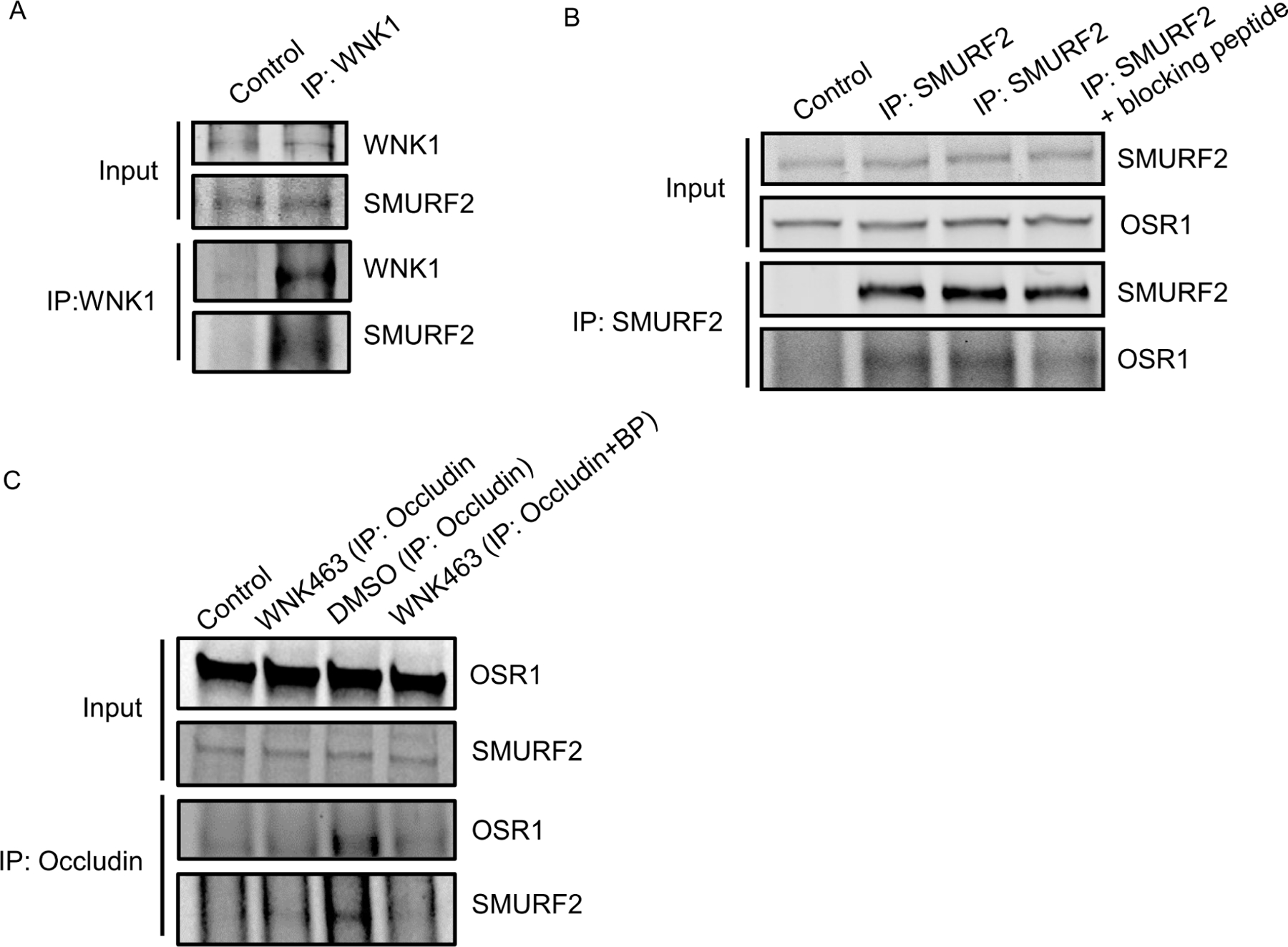
WNK1 kinase activity regulates association of WNK1/OSR1 with SMURF2: (**A**) Representative Western blot showing endogenous co-immunoprecipitation of SMURF2 with WNK1, showing interaction between SMURF2 and WNK1 in HUVECs; *n* = 3. (**B**) Representative Western blot showing endogenous co-immunoprecipitation of OSR1 and SMURF2 in HUVECs which is diminished upon co-incubation with the blocking peptide SAGRRFIVSPVPE (100 μM); *n* = 3. (**C**) Representative Western blot showing endogenous co-immunoprecipitation of OSR1 and SMURF2 with occludin in HUVECs upon treatment with WNK463 (1 μM) which is diminished upon co-incubation with the blocking peptide SAGRRFIVSPVPE (100 μM); *n* = 3.

Given the colocalization between WNK1 and SMURF1/2, we asked whether WNK1 interacts stably with SMURF1/2 in HUVECs and found SMURF2 in endogenous immunoprecipitates of WNK1 (Figure 2A). We found that the WNK1-regulated kinase OSR1 also weakly co-immunoprecipitated with SMURF2 (Figure 2B). OSR1 contains a conserved C-terminal (CCT) domain, which can bind substrates and other proteins via short conserved RFxV motifs [34,35]. A blocking peptide can interfere with OSR1 CCT interactions with RFxV motif-containing proteins such as occludin [10]. We then asked whether the interaction with SMURFs is mediated via the OSR1 CCT domain and SMURF RFxV motifs. We found marginal decreases in OSR1 co-immunoprecipitating with SMURF2 in primary HUVECs upon co-incubation with the blocking peptide (Figure 2B). We previously found that WNK1/OSR1 regulate the turnover of tight junctions [10]. Therefore, we asked whether SMURF2 interacts with the tight junction protein occludin and found that the interaction between occludin and SMURF2 was enhanced upon inhibition of human dermal microvascular endothelial cells (HDMECs) with the pan-WNK inhibitor WNK463 [10]. This interaction was also diminished upon co-incubation with the blocking peptide (Figure 2C). These observations suggest that OSR1 interacts with occludin and weakly with SMURF2 via the CCT domain and that WNK activity regulates the interaction between WNK1/OSR1 and SMURF2.

### 2.3. WNK1/OSR1 Regulates SMURF1/2 and Vice Versa

**Figure 3.**
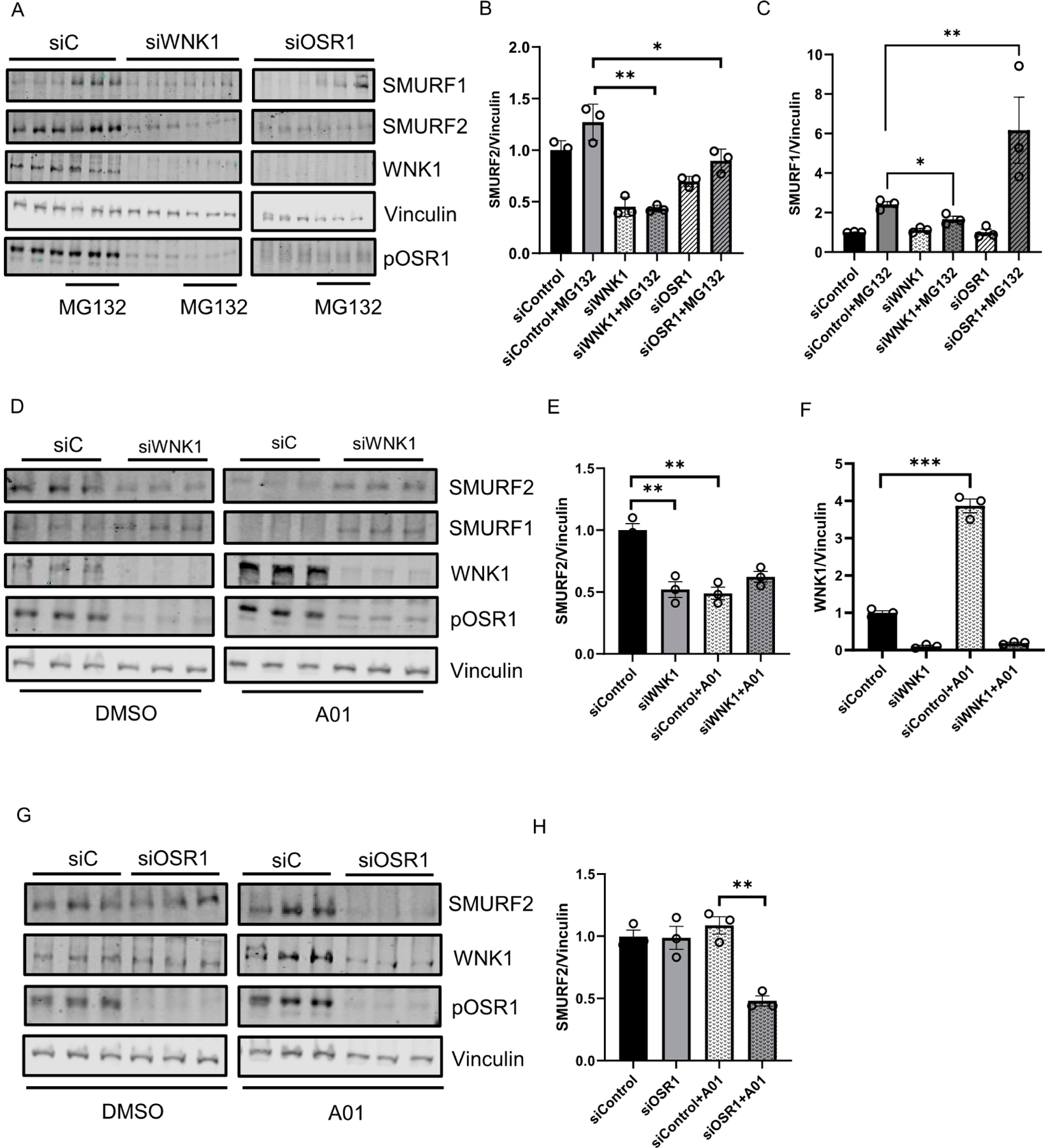
WNK1/OSR1 regulates SMURF1/2 and vice versa: (**A**) Representative Western blot showing SMURF1/2 expression upon WNK1 or OSR1 depletion by siWNK1 or siOSR1, respectively, in HDMECs. It shows an increase in SMURF1 levels upon treatment with proteasomal inhibitor MG132 (10 µM) for 6 h which is prevented upon WNK1 or OSR1 depletion. (**B**) Corresponding quantification of (**A**) showing decreased SMURF2 levels upon WNK1 and OSR1 depletion compared to siControl; *n* = 3. (**C**) Corresponding quantification of (**A**) showing decreased SMURF1 levels upon WNK1 and OSR1 depletion compared to siControl; *n* = 3. (**D**) Representative Western blot showing SMURF1/2 expression upon WNK1 depletion in HDMECs co-treated with SMURF1 inhibitor A01 (2 µM). (**E**) Corresponding quantification of (**D**) showing decreased SMURF2 levels upon WNK1 depletion similar to treatment with the SMURF1 inhibitor A01 (2 µM) alone compared to DMSO or siControl; *n* = 3. (**F**) Corresponding quantification of (**D**) showing increased WNK1 upon treatment with SMURF1 inhibitor A01 (2 µM) alone compared to DMSO or siControl; *n* = 3. (**G**) Representative Western blot showing SMURF1/2 levels upon siRNA depletion of OSR1 in HDMECs. (**H**) Corresponding quantification of (**G**) showing decreased SMURF2 levels with siOSR1 treatment followed by SMURF1 inhibitor A01 (2 µM) treatment overnight compared to siControl or DMSO control; *n* = 3. Data are represented as Mean ± SE; analyzed by unpaired two-tailed Student’s *t*-test or one-way ANOVA. * *p* < 0.05, ** *p* < 0.005, *** *p* < 0.0005.

Given the interaction between WNK1 and SMURFs, we then asked if WNK1 impacts amount of SMURFs. We found that depletion of WNK1 decreased steady state SMURF2 protein expression (Figure 3A,B,D,E). In addition, depletion of WNK1 prevented the expected decrease in steady-state SMURF1 protein. However, upon co-treatment with the proteasomal inhibitor MG132 (10 μM), we observed a decrease in SMURF1 protein expression (Figure 3A,C). Treatment of cells with the SMURF1 inhibitor A01 (2 μM) [36] lead to a significant increase in WNK1 expression (Figure 3D,F). The amount of SMURF2 protein expressed in the absence of added ligands or inhibitors was related to WNK1 expression (Figure 3D,F,G). In contrast, depletion of OSR1 caused no differences in amounts of either SMURF1 or SMURF2 (Figure 3A–C,G,H). Decreased SMURF2 protein expression was observed only in OSR1-depleted cells that were also treated with either the proteasomal inhibitor MG132 or the SMURF1 inhibitor A01 (Figure 3A–C,G,H). In contrast, SMURF1 protein expression increased in OSR1-depleted cells that were also treated with MG132 (Figure 3A,C). These results suggest a complex inter-regulation among WNK1, OSR1 and SMURF1/2.

### 2.4. WNK1 Kinase Activity Mediates SMURF2-Dependent Regulation of ALK5

**Figure 4.**
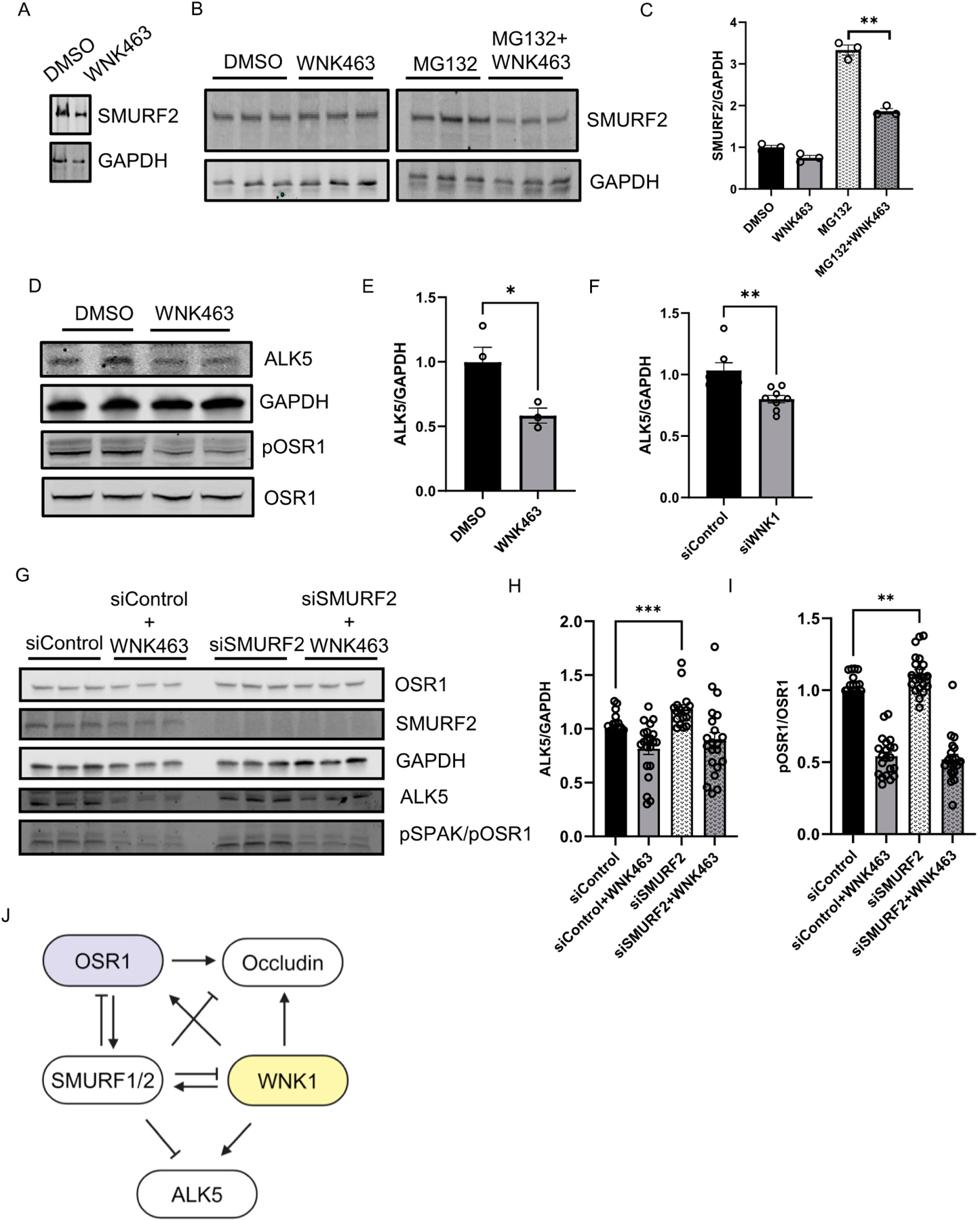
WNK1 kinase activity mediates SMURF2-dependent regulation of ALK5: (**A**) Representative Western blot showing SMURF2 protein levels upon WNK463 overnight treatment (1 μM). (**B**) Representative Western blot showing SMURF2 protein levels upon overnight WNK463 (1 μM) ± MG132 (10 μM). (**C**) Corresponding quantification of (**B**) showing decreased SMURF2 levels upon co-treatment with WNK463 and MG132; *n* = 3. (**D**) Representative Western blot showing ALK5 levels upon DMSO or WNK463 (1 μM) in HDMECs. (**E**) Corresponding quantification of (**D**) showing decreased ALK5 levels upon WNK463 (1 μM) treatment; *n* = 3. (**F**) Quantification of ALK5 in HDMECs treated with siControl or siWNK1; *n* = 6. (**G**) Representative Western blot showing ALK5 and pOSR1 levels upon siControl or siSMURF2 co-treated with DMSO or WNK463 (1 μM) in HDMECs. (**H**) Corresponding quantification of (**G**) showing increased ALK5 levels upon siSMURF2 treatment; *n* = 21. (**I**) Corresponding quantification of (**G**) showing increased pOSR1 levels upon siSMURF2 treatment; *n* = 21. (**J**) Model representing inter-regulation among WNK1, SMURF1/2 and ALK5. Data are represented as Mean ± SE; analyzed by unpaired two-tailed Student’s *t*-test or one-way ANOVA. * *p* < 0.05, ** *p* < 0.005, *** *p* < 0.0005

We asked whether the kinase activity of WNK1 is important for regulation of SMURF2 and found that treatment with WNK463 decreased SMURF2 protein expression, similar to the effect of WNK1 knockdown (Figure 4A). We also found that baseline SMURF2 protein expression was enhanced by treatment with the proteasomal inhibitor MG132; WNK463 co-treatment efficiently decreased SMURF2 protein expression (Figure 4B,C). These results suggest that the observed effects on SMURFs are dependent on WNK1 activity. Previously, we found that WNK463 decreased expression of the type 1 TGF-β receptor ALK1 [10]. Degradation of the TGF-β type I receptor, ALK5, is facilitated via recruitment of the SMURF2 complex [12,13,21,22]. Given the regulation of WNK1 by SMURF2 and effects of WNK inhibition of ALK1, we asked whether WNK1 kinase activity also regulates ALK5. We found that treatment with WNK463 did, in fact, decrease ALK5 protein expression as well (Figure 4D,E). We also found that knockdown of WNK1 decreased SMURF2 protein expression (Figure 4F). As expected, knockdown of SMURF2 increased ALK5 protein expression (Figure 4G,H). Interestingly, SMURF2 knockdown also resulted in a corresponding increase in the phosphorylated active form of OSR1, suggestive of enhanced WNK1 activity (Figure 4G,I). Given the regulation of ALK5 by WNK1, and that of WNK1 by SMURF2, these data suggest that the increase in ALK5 protein expression by SMURF2 knockdown may, in part, result from increased WNK1 activity. Overall, these results suggest that WNK1 kinase activity and SMURF1/2 reciprocally regulate each other to affect responses to TGF-β (Figure 4J).

## 3. Discussion

Unanticipated findings revealed that WNK1 and SMURFs reciprocally regulate each other to fine-tune TGF-β signaling. Aggregated structures containing SMURF1/2 and WNK1 in our study are similar to those observed with respect to SMURF2, and clustering of SMURF2 is suggested to regulate its E3 ubiquitin ligase activity. [12,19]. We show that the depletion or inhibition of WNK1 decreases SMURF2. The amount of SMURF2 protein present upon the inhibition of SMURF1 is dependent on WNK1 protein amount. One possible explanation is that SMURF1 regulates SMURF2 protein, and this is dependent on the relative expression of WNK1. SMURF2 inhibits its own ubiquitinase activity and is thereby stabilized. Therefore, it is possible that the inhibition of WNK1 may enhance the activity of SMURF1/2 and thereby lead to the downregulation of itself and ALK5, as observed in this study. Future studies will focus on addressing the mechanistic details of this potential mode of regulation.

Our study reports that WNK1 colocalizes with SMURF1/2 in large punctate structures. SMURF1 mediates p62 biomolecular condensation to promote autophagy of NRF2, a protein that is known to interact with WNK1 to enhance cellular oxidative response [37,38]. However, we did not find the co-localization of p62 in these WNK1-SMURF1/2 large punctate structures. The WW domains of SMURF/NEDD4 E3 ligases generally recognize and bind proline-rich sequences such as PY motifs on substrate proteins [19]. Interestingly, PY motifs are also found in WNK1. The activity of E3 ligases such as SMURFs is regulated by clustering of the PY motifs on their substrates [19]. WNK1 has been reported to form punctate structures referred to as WNK bodies which are membraneless cytosolic signaling foci that sequester WNKs. These structures have been reported in kidney epithelial cells during changes in total body potassium balance [25]. Therefore, we speculate that these large punctate structures we observed in our study may be similar to the WNK bodies and that such clustering of WNK1 may regulate the activity of SMURFs to affect TGF-β signaling output. Nevertheless, this interesting hypothesis requires further examination.

We found that SMURF2 interacts with OSR1 via its conserved C-terminal (CCT) domain. The CCT domain of OSR1 binds substrates and other proteins via short conserved RFxV motifs [34,35]. We had previously reported that OSR1 CCT interacts with the RFxV motif containing proteins such as occludin [10]. We found that SMURF2 interacts with occludin and this interaction was enhanced upon inhibition with the pan-WNK inhibitor WNK463 [10], but decreased upon co-incubation with the CCT blocking peptide. These observations suggest that WNK activity regulates the interaction between WNK1/OSR1, occludin and SMURF2. We propose that the CCT of OSR1 mediates the interaction between SMURF2 and occludin. SMURF2 has been shown to be in a complex with occludin during TGF-β signaling [22], but the mechanistic significance of interaction between SMURF2 and occludin remains to be understood.

Signal transduction pathways regulated by TGF-β control a diverse array of cellular processes such as vascular remodeling [39]. Depending on the cellular context, the TGFβ signaling pathway exhibits differential and opposing responses [39–42]. This occurs via complex regulatory mechanisms and crosstalk among several TGF-β mediators [39–42]. The duration and amplitude of TGF-β signaling are tightly controlled by various proteasome-mediated degradation mediators involving E3 ubiquitin ligases such as SMURF1/2, among others [39–42]. These observations suggest that the dynamic convergence of inputs from WNK1 to SMURFs affects TGF-β receptors, essential SMAD transcription factors and tight junction components to fine-tune TGF-β signaling to facilitate diverse cellular outcomes. Therefore, we propose that the expression and activity of WNK1 contribute to the context-specificity in TGF-β signaling.

## 4. Methods

### 4.1. Cell Lines

Human Umbilical Vein Endothelial Cells (HUVECs: ATCC, Manassas, VA, USA; PCS-100-013) were grown in a complete VascuLife^®^ EnGS media kit (Fisher Scientific, Waltham, MA, USA; 50-311-891) supplemented as per the manufacturer’s instructions. In experiments requiring knockdowns, Human Dermal Microvascular Endothelial Cells (HMEC-1: ATCC, USA; CRL-3243) were used. These cells were grown in complete MCDB media (Fisher Scientific, USA; MT15100CV) supplemented with 10% fetal bovine serum (Sigma Aldrich, St. Louis, MA, USA; F0926), 1% L-glutamine, 1% penicillin and streptomycin, 1 µg/mL hydrocortisone (Sigma Aldrich, USA; H0888 or H6909), and 10 ng/mL epidermal growth factor (EGF: Cell Signaling Technology, Danvers, MA, USA; 8916SC). All cells were maintained at 37 °C and 5% CO_2_.

### 4.2. Co-Immunoprecipitation

Cells were lysed in 1X lysis buffer (50 mM HEPES, 0.1 M NaCl, 0.5 mM EDTA, 0.1% SDS) supplemented with protease inhibitor cocktail, PMSF and phosphatase inhibitors (PhosStop). Cell extracts were harvested and cleared by centrifugation. 1X IP buffer (50 mM HEPES, 0.1 M NaCl, 0.5 mM EDTA, and 1% CHAPS (Sigma Aldrich, USA; C3023) supplemented with protease inhibitor cocktail, PMSF, and phosphatase inhibitors (Sigma Aldrich, USA; 4906837001) was added in a 2:1 ratio to the cell lysate. Samples were incubated with primary antibody (control sample incubated with rabbit IgG primary antibody: 1:100) overnight at 4 °C and then with Protein A/G PLUS-Agarose (Santa Cruz Biotechnology, Dallas, TX, USA; sc-2003) beads for 1 h with head-to-tail rotation. This was performed either in the absence or presence of CCT blocking peptide SAGRRFIVSPVPE (100 µM) (United Biosystems, Herndon, VA, USA). Samples were then washed three times with 1X IP buffer before adding 5X SDS buffer (0.25% bromophenol blue, 0.5 M DTT, 50% glycerol, 10% SDS, 0.25 M Tris-Cl) and heating at 90 °C for 2 min. Samples were then run on 4–20% Mini-PROTEAN^®^ TGX^™^ Precast Protein Gels (Bio-Rad, Hercules, CA, USA; 4568096) or 12% polyacrylamide home-made gels before being transferred to PVDF membranes. Membranes were then washed in TBS-T before being blocked with TBS-based blocking buffer (LI-COR, Lincoln, NE, USA). Membranes were incubated with primary antibodies and then washed again before being incubated with species-specific, light chain-specific secondary antibodies (Jackson ImmunoResearch Labs, West Grove, PA, USA; 115-655-174 and 211-622-171) and imaged using LI-COR imaging.

### 4.3. Immunofluorescence

Cell lysates containing 1× SDS buffer were homogenized with a 27-G syringe, and whole lysates were run on 4–20% Mini-PROTEAN^®^ TGX^™^ Precast Protein Gels (Bio-Rad, USA; 4568096) or 6/10/12% home-made polyacrylamide gels before being transferred to PVDF membranes (Bio-Rad, USA; 1620177). Membranes were then washed in TBS-T before being blocked with TBS-based blocking buffer (LI-COR). Membranes were incubated with primary antibodies and then washed again before being incubated with species-specific secondary antibodies and imaged and quantified using LI-COR.

### 4.4. siRNA Knockdown

Oligonucleotides encoding siRNA for human WNK1 (siWNK1: 5′ CAGACAGUGCAG UAUUCACTT 3′), control siRNA (Thermo Fisher Scientific, Nashik, MA, USA; 4390844) as in [9], OSR1 siRNA (Thermo Fisher Scientific, USA; s19303 Silencer^®^ Select), and SMURF2 siRNA (sc-41675, Santa Cruz Biotechnology, USA) were used. HDMEC cells were transfected with 20 nM siRNA using Lipofectamine RNAiMax reagent (Thermo Fisher Scientific, USA; 13778150). After 24–72 h of transfection, cells underwent their respective treatments and were then harvested in 1X SDS buffer (0.05% bromophenol blue, 0.1 MDTT, 10% glycerol, 2% SDS, and 0.05 M Tris-Cl) with 5% β-mercaptoethanol.

### 4.5. Immunoblotting

Cell lysates containing 1X SDS buffer were homogenized with a 27-G syringe, and whole lysates were run on 4–20% Mini-PROTEAN^®^ TGX^™^ Precast Protein Gels (Bio-Rad, USA; 4568096) or 6/10/12% home-made polyacrylamide gels before being transferred to PVDF membranes (Bio-Rad, USA; 1620177). Membranes were then washed in TBS-T before being blocked with TBS-based blocking buffer (LI-COR). Membranes were incubated with primary antibodies and then washed again before being incubated with species-specific secondary antibodies and imaged and quantified using LI-COR.

### 4.6. Reagents

WNK463 (Selleck Chemicals, Houston, TX, USA; S8358), SMURF1 inhibitor A01 (Sigma Aldrich, USA; SML1404), MG132 (Sigma Aldrich, USA; M7449), TGF-β1 (Cell Signaling Technology, USA; 8915LC), anti-Vinculin antibody (Sigma Aldrich, USA; V9131), anti-pOSR1 antibody (EMD Millipore, Billerica, MA, USA; 07–2273), anti-OSR1 polyclonal antibody (Cell Signaling, Danvers, MA, USA; 3729S), anti-OSR1 monoclonal antibody (VWR, Radnor, PA, USA; 10624-616), anti-WNK1 antibody (Cell Signaling, USA; 4979S), anti- GAPDH antibody (Cell Signaling Technology, USA; 97166L), anti-SMURF1 antibody (Santa Cruz Biotechnology, USA; sc-100616), anti-SMURF2 antibody (Santa Cruz Biotechnology, USA; sc-393848), anti-flag antibody (Sigma-Aldrich, Saint Louis, MO, USA; F1804) and Q256 WNK1 antibody were homemade as in [7], and Optimem (Invitrogen, Carlsbad, CA, USA; 51985-034), Lipofectamine 2000 (Life Technologies, Carlsbad, CA, USA; 11668019), bumetanide (Sigma Aldrich, USA; B3023) and 96-well plates (Corning, Steuben County, NY, USA; 3904 or Greiner, 655090) were used.

### 4.7. Statistics and Reproducibility

The data are presented mean ± SEM from at least three independent experiments. All presented micrographs (immunofluorescence images) are representative images from three representative experiments as indicated in the figure legends. For the quantification of immunofluorescence images, the number of cells used for each representative experiment is indicated and *p* values between two groups were determined using unpaired t-tests. Results are expressed as mean ± SEM. Single intergroup comparisons between 2 groups were performed with 2-tailed Student’s *t*-test as specifically mentioned in each case. *p* < 0.05 was considered statistically significant.

## Data Availability

The data presented in this study are available upon request from the corresponding author. No new data set was created in this study. We will follow all NIH policies with respect to sharing reagents, materials and information with other investigators. Detailed protocols will be provided to anyone who requests them. Upon publication, this manuscript will be submitted to the National Library of Medicine’s PubMed Central as outlined by NIH policy.
